# *In vitro* maturation 2.0: a new era for an underdog in ART

**DOI:** 10.5935/1518-0557.20250177

**Published:** 2026

**Authors:** Marcos Iuri Roos Kulmann, Gabriella Mamede Andrade, Adriana Bos-Mikich, Norma Pagnoncelli de Oliveira, Marcelo Ferreira, Nilo Frantz

**Affiliations:** 1 Nilo Frantz Medicina Reprodutiva, Porto Alegre, Brazil; 2 Departamento de Ciências Morfológicas, Instituto de Ciências Básicas da Saúde, Universidade Federal do Rio Grande do Sul, Porto Alegre, Brazil

**Keywords:** *In Vitro* Maturation, meiotic maturation, cytoplasmic maturation, patient-friendly

## Abstract

*In vitro* maturation (IVM) of human oocytes, once a pioneering concept predating conventional IVF, has long remained an underutilized technique in assisted reproduction. Despite early promise, clinical adoption of IVM has been limited due to lower embryo developmental competence and live birth rates compared to IVF. However, recent advances in the understanding of oocyte physiology, including cumulus-oocyte communication, the regulatory roles of cAMP/cGMP signaling pathways, and endocrine modulation of meiotic resumption, have reignited interest in optimizing IVM protocols. Innovations such as biphasic Capacitation (CAPA) IVM systems and the use of ovarian somatic support cells (OSCs) derived from induced pluripotent stem cells (iPSCs) aim to replicate the dynamic follicular environment more accurately and enhance oocyte competence. Clinical studies suggest that, while IVM still results in modestly lower cumulative live birth rates compared to conventional IVF, it offers significant advantages for selected patient populations, particularly women with polycystic ovary syndrome (PCOS), high ovarian reserve, or those requiring fertility preservation. Importantly, current evidence supports the genetic and epigenetic safety of IVM-derived offspring. As technical refinements continue and professional education expands, IVM is poised to fulfill its potential as a safer, less invasive, and more accessible alternative within the landscape of assisted reproductive technologies.

## INTRODUCTION

**Figure 1:**
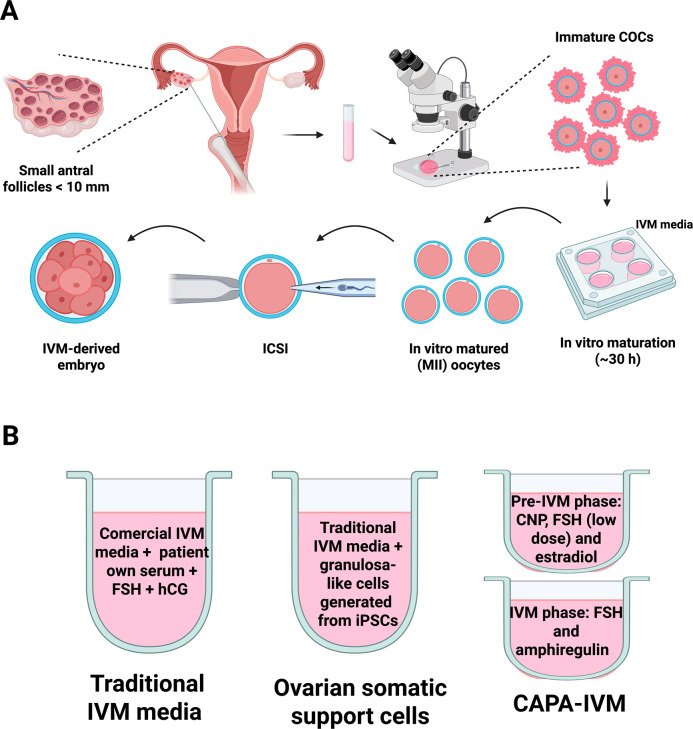
In vitro maturation of human oocytes. A) Workflow for the IVM treatment. B) Media composition for different IVM systems. Created in BioRender. Roos Kulmann, M. I. (2025) https://BioRender.com/31kaba0.

*In Vitro* Maturation (IVM) is an alternative Assisted Reproductive Technology (ART), traditionally defined as the maturation of immature oocytes *in vitro* from the germinal vesicle (GV, prophase I) to the metaphase II (MII) stage (Fig. 1A). These oocytes are typically retrieved from mid-sized antral follicles in unstimulated ovaries. Over time, IVM protocols have evolved to include minimal stimulation with follicle-stimulating hormone (FSH) and/or human chorionic gonadotropin (hCG). In recognition of its clinical value, the American Society for Reproductive Medicine (ASRM) declared IVM a non-experimental technique in 2021 (Practice Committees of the ASRM, 2021), especially applicable to women with polycystic ovaries (PCO), polycystic ovary syndrome (PCOS), and those requiring fertility preservation, such as cancer patients.

While commonly associated with these populations, IVM may also be suitable for normo-ovulatory patients with high ovarian reserve. Although initially perceived as a strategy to avoid ovarian hyperstimulation syndrome (OHSS), a complication now largely mitigated by the use of GnRH antagonists (Mills & Dahan, 2022), IVM offers additional benefits. It represents a more patient-friendly alternative to conventional in vitro fertilization (IVF), which requires controlled ovarian stimulation with high-dose gonadotropins to collect multiple mature (MII) oocytes. By reducing or eliminating the need for ovarian stimulation, IVM can lower treatment costs (Braam *et al.*, 2021), minimize the number of clinic visits and ultrasound monitoring, and reduce medication-related side effects (Marchante *et al.*, 2024), factors that often contribute to treatment discontinuation.

The concept of IVM was first demonstrated in rabbits (Pincus & Enzmann, 1935) and subsequently in other mammalian species, including humans (Edwards, 1965). In the late 1960s, Robert Edwards, widely regarded as a pioneer in reproductive medicine, initially explored IVM as a treatment for infertility (Edwards *et al.*, 1969), before shifting his focus toward developing IVF. Reflecting on the field trajectory decades later, Edwards wrote in a 2007 publication: “With hindsight, should we have developed IVM rather than ovarian stimulation in 1969?” (Edwards, 2007).

Although IVF went on to become a mainstream technique, IVM remained on the sidelines for decades. The first live birth following IVM was reported in 1991 (Cha *et al.*, 1991), and the first successful IVM pregnancy in a PCO patient was documented in 1994 (Trounson *et al.*, 1994). In Latin America, the first IVM birth occurred in 2008 (Frantz *et al.*, 2008). Despite its early origins, even predating IVF, IVM clinical development has been remarkably slow. To date, only an estimated 5,000-6,000 babies have been born worldwide using IVM (Das & Son, 2023), compared to millions born through IVF.

This limited adoption may be attributed to several factors, including lower embryo developmental potential in early IVM protocols and the technical complexity of the procedure. Additionally, the development of commercially available IVM systems did not keep pace with recent scientific discoveries on the physiology of oocyte maturation *in vivo*. As a result, IVM has consistently demonstrated lower success rates compared to conventional IVF (Vuong *et al.*, 2020) and remains largely confined to a few specialized centers worldwide.

Recent scientific advances and the development of innovative culture systems have reignited interest in IVM, offering promising opportunities to broaden access to ART and enhance clinical outcomes. Key innovations include biphasic IVM systems designed to preserve oocyte-cumulus communication (Sanchez *et al.*, 2019), the addition of granulosa-like cells (Piechota *et al.*, 2023) or follicular fluid-derived extracellular vesicles (EVs) (Makieva *et al.*, 2025) to better support oocyte maturation *in vitro*, and microfluidic chips (Zargari *et al.*, 2016). Could this new era of IVM represent the next breakthrough in ART, positioned as a gentler alternative between intrauterine insemination (IUI) and conventional IVF? This review explores this possibility by focusing on molecular mechanisms underlying physiological oocyte maturation, current IVM clinical protocols, their safety, and whether IVM still lags behind standard IVF treatment in terms of clinical efficacy.

## PHYSIOLOGY OF OOCYTE MATURATION

Oocyte maturation is a finely regulated physiological process that begins during fetal development and spans decades, culminating in the resumption of meiosis just prior to ovulation (Jamnongjit & Hammes, 2005). The initiation of meiosis during fetal life results in the formation of primordial follicles, wherein oocytes become arrested in prophase I, also known as the GV stage, surrounded by a single layer of flattened pre-granulosa cells (as reviewed in Telfer *et al.*, 2023). This arrest is maintained until the follicle is recruited for growth and maturation, a process that remains tightly coordinated by the somatic compartment of the follicle.

The maintenance of meiotic arrest in fully grown oocytes is primarily governed by high levels of intracellular cyclic adenosine monophosphate (cAMP) (Cho *et al.*, 1974), which inhibits the maturation-promoting factor (MPF) via activation of cAMP-dependent protein kinase A (PKA) (Shitsukawa *et al.*, 2001). Two sources contribute to intra-oocyte cAMP levels: (1) endogenous production within the oocyte (Mehlmann *et al.*, 2002), and (2) transzonal flux of cAMP from surrounding cumulus cells (CCs) under the influence of FSH (Anderson & Albertini, 1976). Critically, cyclic guanosine monophosphate (cGMP), synthesized in granulosa and CCs under the regulation of C-type natriuretic peptide (CNP), diffuses through gap junctions to the oocyte and inhibits phosphodiesterase 3A (PDE3A) (Zhang *et al.*, 2010a; Tsuji *et al.*, 2012), thereby preventing cAMP hydrolysis and reinforcing meiotic arrest (Norris *et al.*, 2009). Therefore, maintaining the communication with CCs is crucial for oocyte meiotic arrest.

FSH plays a central role in promoting folliculogenesis and oocyte growth by acting on its receptor, expressed in granulosa cells from early/mid antral stage (Casarini *et al.*, 2022) and possibly in the oocyte (Méduri *et al.*, 2002). FSH drives granulosa cell proliferation, steroidogenesis, and antrum formation, eventually leading to the differentiation in mural granulosa cells (MGCs) and CCs (Eppig, 2001). The latter remain in intimate communication with the oocyte via transzonal projections (TZPs) and gap junctions, facilitating the bidirectional exchange of signals and metabolites (Kidder & Mhawi, 2002). During the growth phase, the oocyte increases in diameter and accumulates essential organelles, mRNAs, and proteins, collectively referred to as cytoplasmic maturation, largely supported by the surrounding CCs. Notably, the oocyte actively regulates CC function through the secretion of oocyte-secreted factors (OSFs), including growth differentiation factor 9 (GDF9) and bone morphogenetic protein 15 (BMP15) to modulate CC proliferation, metabolic activity, inhibition of premature luteinization, and responsiveness to meiotic inducers (Gilchrist, 2011).

Meiotic resumption is triggered by the preovulatory surge of luteinizing hormone (LH), which does not act directly on the oocyte or CCs, both of which normally lack LH receptors before ovulation (Diaz *et al.*, 2007), but instead induces the expression of epidermal growth factor (EGF)-like peptides (amphiregulin, epiregulin, and betacellulin) in MGCs (Park *et al.*, 2004). These peptides activate EGF receptors on CCs, a receptor system whose functional maturation depends on FSH signaling in concert with OSFs (Fan *et al.*, 2009). The resulting signaling cascade likely leads to cumulus expansion, retraction of TZPs, and gap junction breakdown, culminating in a rapid decrease in intra-oocyte cGMP. This decline lifts the inhibition on PDE3A, leading to cAMP degradation, MPF activation, and germinal vesicle breakdown (GVBD) (Gilchrist, 2011).

The physiological coordination of meiotic arrest and resumption is lost when the oocyte is removed from its follicular niche, as occurs in conventional IVM protocols (Gilchrist & Smitz, 2023), resulting in spontaneous and asynchronous nuclear maturation. This unphysiological resumption of meiosis is hypothesized to compromise developmental competence by bypassing essential cytoplasmic and nuclear maturation events.

Understanding the molecular orchestration of oocyte maturation *in vivo* provides critical insights for designing culture systems that can faithfully mimic these conditions.

## A CLINICAL PERSPECTIVE ON IVM

### Patient selection

IVM was initially proposed for patients with PCOS or PCO-like conditions who face an elevated risk of developing OHSS during controlled ovarian stimulation. However, with the widespread use of GnRH antagonist triggers, the risk of OHSS has significantly reduced. As a result, the scope of IVM has broadened to include other patient groups, such as egg donors (Holzer *et al.*, 2007), women with ovarian resistance to FSH (Galvão *et al.*, 2018) and those requiring fertility preservation due to gonadotoxic treatments like cancer therapies (Cao & Chian, 2009; Huang *et al.*, 2010; Chian *et al.*, 2013) (Practice Committees of the ASRM, 2021).

A major advantage of IVM lies in its potential to reduce treatment costs compared to IVF cycles. Cost-effectiveness analyses suggest that IVM can decrease expenses related to gonadotropin use, office visits, and ultrasound examinations, leading to an estimated 34% reduction in overall treatment costs (Braam *et al.*, 2021). As a result, the implementation of an IVM program could significantly expand access to ART for patients who might otherwise be unable to afford them, particularly in areas where IVF costs are high and not covered by insurance. Additionally, many patients may choose IVM to avoid the physical and emotional side effects associated with the high doses of gonadotropins, making IVM a more comfortable and less stressful alternative. In fact, women undergoing minimal gonadotropin priming during IVM have reported experiencing fewer side effects, such as pain, nausea, bleeding, and breast swelling, compared to those undergoing conventional controlled ovarian stimulation for IVF (Marchante *et al.*, 2024). This improved patient experience adds to the appeal of IVM as a less invasive and more patient-friendly option.

### IVM definitions

There are varying definitions and approaches to IVM. A more traditional or “purist” view of IVM involves the retrieval of COCs containing GV oocytes without any prior gonadotropin stimulation (Gilchrist & Smitz, 2023). However, some groups have suggested that using gonadotropin priming, administering FSH, hCG, or both, could improve pregnancy rates in IVM treatments (Fadini *et al.*, 2009).

In standard IVF cycles, some of the collected oocytes are immature, typically at the GV or MI stages. Despite losing communication with CCs after denudation, these oocytes often undergo spontaneous maturation *in vitro* without the need for specialized IVM media or stimulation, a process referred to as Rescue IVM (Coticchio *et al.*, 2025). Clinically, these oocytes can serve as surplus, potentially enhancing cumulative pregnancy rates, especially in patients with poor prognosis (Liu *et al.*, 2020; Coticchio *et al.*, 2025). It is important to distinguish this form of rescue maturation from the conventional IVM approach, which typically involves minimal or no gonadotropin stimulation.

### FSH and hCG priming

Human follicles measuring 2-6 mm in diameter are known to express high levels of FSH receptors, and when activated by FSH, this promotes follicular growth and estradiol production (Jeppesen *et al.*, 2012; Kristensen *et al.*, 2018; Gilchrist & Smitz, 2023). Various regimens have been suggested in the literature, including cumulative doses of 450 to 600 IU of FSH administered over 2 to 5 days, typically starting on day 2 or 3 of the menstrual cycle (Wynn *et al.*, 1998; De Vos *et al.*, 2011). The effectiveness of FSH priming without hCG in IVM cycles remains a topic of debate. Junk *et al.* (2003) reported improved maturation rates with FSH priming but no significant impact on embryonic development. Since FSH priming does not trigger meiotic resumption in oocytes *in vivo*, immature compact COCs are collected during oocyte retrieval (Das & Son, 2023).

Interestingly, granulosa cells from antral follicles smaller than 6 mm do not usually express LH receptors, making the benefits of hCG priming questionable (Jeppesen *et al.*, 2012; Gilchrist & Smitz, 2023). In practice, hCG priming may primarily promote the maturation of dominant follicles within the cohort, leading to a mixture of asynchronous mature and immature oocytes at collection, which complicates the strict definition of IVM. Similar to FSH priming, studies have demonstrated that hCG priming alone can increase oocyte maturation rates but does not necessarily improve pregnancy outcomes (Chian *et al.*, 2000; Junk *et al.*, 2003; Zheng *et al.*, 2012; Lin *et al.*, 2020).

The debate extends to the combined use of FSH and hCG priming. Fadini *et al.* (2009) showed that combining FSH and hCG priming significantly increased pregnancy rates (29.9%) compared to unstimulated cycles (15.3%), FSH priming alone (17.3%), or hCG priming alone (7.6%). However, Lin *et al.* (2003) reported that adding FSH priming to hCG priming did not result in any noticeable improvements in pregnancy rates. Therefore, further research is needed to determine whether gonadotropin priming can provide additional benefits to patients undergoing IVM treatment.

### Oocyte retrieval

There is no consensus regarding the ideal timing for oocyte retrieval in IVM cycles. However, most studies suggest selecting a lead follicle diameter of up to 10 mm, based on findings by Cobo *et al.* (1999), which demonstrated that oocyte retrieval at this stage resulted in a higher number of recovered oocytes and improved blastocyst rates compared to retrieval when the lead follicle exceeded 10 mm. Larger follicles may induce atresia in sibling oocytes, potentially compromising their quality and development. Typically, the oocyte retrieval is scheduled 42-46 h after the last FSH injection in FSH-primed cycles (De Vos *et al.*, 2011; Gilchrist *et al.*, 2024), or 36-38 hours after administration in hCG-primed cycles (Zheng *et al.*, 2012).

Compared to IVF, oocyte retrieval in IVM presents additional complexities due to the smaller follicular size (typically 2 to 6 mm), lack of CCs expansion and tighter adherence of immature oocytes to the follicle wall, requiring a more delicate and technically demanding aspiration process (Practice Committees of the ASRM, 2021). The retrieval procedure is more time-consuming, often taking twice as long as conventional IVF, as precise handling is necessary to optimize oocyte recovery while minimizing follicular collapse.

The choice of aspiration needle plays a crucial role in IVM success. While a single-lumen 17-gauge needle commonly used in IVF can be utilized, many centers opt for finer-gauge (19-21G) double-lumen needles (Wynn *et al.*, 1998; Yan *et al.*, 2021). These finer needles enhance precision when aspirating smaller follicles and reduce trauma to the follicular environment. Additionally, aspiration pressure must be carefully controlled to preserve COCs. Lower pressures, typically between 80 and 120 mmHg, are recommended to minimize shear stress and prevent dissociation of CCs from the oocyte, thereby maintaining its developmental competence in subsequent *in vitro* maturation steps (Hashimoto *et al.*, 2007).

## IVM IN THE LABORATORY

### The IVM media

Early clinical findings revealed that oocytes matured *in vitro* did not exhibit comparable developmental competence to oocytes matured *in vivo* (Chian, 2004). This discrepancy has sparked global research efforts to optimize IVM. However, progress has been exceedingly slow. Commercially available systems have failed to keep pace with recent scientific advances, largely due to the limited interest of IVF clinics in adopting this alternative ART. As a result, a fully effective IVM protocol that accurately replicates *in vivo* maturation remains elusive.

Currently available IVM systems are relatively basic, typically relying on the addition of FSH and hCG to induce maturation (Fig. 1B). However, as previously discussed, the LH surge does not usually act directly on CCs or the oocyte. Instead, it initiates a signaling cascade leading to the expression and secretion of EGF-like peptides, such as amphiregulin, epiregulin, and betacellulin in MGCs. These peptides are promising additions to IVM media, as they promote meiotic resumption in a more physiological manner. In a murine IVM model, EGF-p supplementation led to improved blastocyst formation rates and embryo quality compared to FSH or EGF alone (Richani *et al.*, 2013).

EGF-p exert their effects through activation of EGF receptor, whose expression is supported by FSH, GDF9 and BMP15. Supplementation of GDF9 and BMP15 has also been shown to enhance blastocyst and live birth rates in bovine (Hussein *et al.*, 2006) and murine (Yeo *et al.*, 2007) IVM models, respectively. In parallel, emerging IVM strategies highlight the importance of maintaining cumulus-oocyte communication to support cytoplasmic maturation, avoiding premature TZP retraction and meiosis resumption. In this sense, CNP plays a critical role in maintaining intra-oocyte cAMP levels and prophase I arrest (Zhang *et al.*, 2010a), while the expression of its receptor in CCs is supported by estradiol (Zhang *et al.*, 2011), a hormone that has been shown to enhance oocyte competence in multiple IVM models (Zheng, 2003; Maksura *et al.*, 2021).

Collectively, these findings suggest that an improved baseline IVM medium should incorporate EGF-like peptides, FSH, GDF9, BMP15, and estradiol to better replicate *in vivo* signaling and support oocyte competence.

### New IVM systems

In an effort to overcome the suboptimal outcomes typically observed in IVM cycles, new IVM systems have been developed. Capacitation IVM (CAPA-IVM) is an emerging approach designed to preserve oocyte-cumulus communication by preventing premature maturation and TZP retraction, both of which are critical for cytoplasmic maturation and enhanced developmental potential (Romero *et al.*, 2016; Sanchez *et al.*, 2019). Spontaneous oocyte maturation occurs primarily due to a decline in intracellular cAMP. While cAMP is sustained through endogenous oocyte production and FSH-stimulated synthesis in CCs, it also depends on cGMP, which inhibits PDE3 and prevents cAMP degradation (Degerman *et al.*, 1997).

As previously mentioned, cGMP production in CCs is stimulated by CNP secreted by MGCs (Zhang *et al.*, 2010a). Following IVM COC retrieval, this source of CNP is lost, leading to a drop in cGMP levels and a consequent decline in cAMP within the oocyte, triggering premature meiotic resumption (Degerman *et al.*, 1997). Capacitation IVM (CAPA-IVM) mitigates this issue by introducing a pre-IVM phase with exogenous CNP (Gilchrist *et al.*, 2024). The rationale for this supplementation is to sustain cGMP levels, stabilize intra-oocyte cAMP, and delay nuclear maturation, thereby allowing additional time for cytoplasmic maturation (Sanchez *et al.*, 2019).

CAPA-IVM has demonstrated superior efficiency compared to commercially available IVM systems, leading to higher oocyte maturation rates, increased yield of good-quality embryos, and improved clinical pregnancy outcomes (Gilchrist *et al.*, 2024). The first randomized controlled trial (RCT) comparing CAPA-IVM with conventional IVF found that CAPA-IVM was only slightly inferior in terms of Day-3 embryo quality and cumulative pregnancy rates (Vuong *et al.*, 2020). Importantly, studies assessing the safety of CAPA-IVM have shown no significant differences in DNA methylation (Saenz-de-Juano *et al.*, 2019) and health of children (Vuong *et al.*, 2022).

Another innovative approach to IVM involves co-culturing cumulus-oocyte complexes (COCs) with ovarian somatic support cells (OSCs) generated from human-induced pluripotent stem cells (iPSCs). These OSCs, differentiated through the expression of specific transcription factors, exhibit granulosa-like characteristics (FOXL2+, AMHR2+, NR2F2+) (Pierson Smela *et al.*, 2023) and may restore critical signaling from mural granulosa cells (MGCs) within the IVM culture. Upon FSH stimulation, OSCs produce growth factors and steroids, potentially recreating key elements of the follicular microenvironment (Pierson Smela *et al.*, 2023).

As previously discussed, the preovulatory LH surge stimulates MGCs to produce EGF-like peptides, amphiregulin, epiregulin, and betacellulin, which activate EGF receptors in both an autocrine manner (within MGCs) and a paracrine manner (in CCs) (Park *et al.*, 2004). This cascade triggers meiotic resumption via the ERK1/2 pathway. While not yet fully characterized, OSCs may also express CNP, helping sustain intra-oocyte cAMP levels and maintaining cumulus-oocyte communication.

Unlike conventional static culture media, OSCs dynamically respond to co-culture signals, potentially fine-tuning their gene expression in response to cues from CCs and the oocyte (Paulsen *et al.*, 2024). Notably, OSC-assisted IVM has been shown to generate oocytes with transcriptomic profiles more closely resembling those of *in vivo*-matured oocytes (Paulsen *et al.*, 2024). Furthermore, this approach has resulted in higher MII maturation and euploid blastocyst formation rates in comparison to commercially available IVM systems (Piechota *et al.*, 2023). Future clinical data on pregnancy outcomes will determine whether OSC-IVM significantly enhances success rates in IVM cycles.

Although additional laboratory procedures in IVM cycles are well established, the technique complexity may require a larger and more experienced team of embryologists. Oocyte retrieval from antral follicles is technically challenging due to the smaller size of COCs, increased blood content in follicular fluid, and the need for additional sieves to aid in oocyte capture (Practice Committees of the ASRM, 2021). Moreover, hCG priming can cause oocyte asynchrony, leading to a mixture of maturation stages at the time of collection (Gilchrist & Smitz, 2023). In that case, COC morphology can help infer oocyte maturity: expanded COCs should be denuded and MII oocytes inseminated immediately, while unexpanded ones are directed to IVM culture. This results in staggered insemination times for oocytes from the same patient, often at inconvenient hours, thereby increasing the overall workload in the laboratory.

## IVM *VERSUS* IVF: HOW FAR ARE WE?

Multiple studies have investigated whether IVM offers comparable efficiency to IVF, particularly in women with PCO/PCOS. Initial research began with case-control studies and later progressed to RCTs. Child (2002) conducted the first case-control study comparing unstimulated IVM with IVF in age-matched PCOS patients. The study reported clinical pregnancy and live birth rates per oocyte retrieval of 26.2% and 15.9%, respectively, in IVM cycles, compared to 38.3% and 26.2% in IVF cycles (Table 1). Although these differences were not statistically significant, there was a trend towards lower success rates with IVM. Furthermore, 11.2% of patients undergoing IVF developed moderate or severe OHSS, while none of the IVM patients experienced this complication. Similarly, a retrospective case-control study by Gremeau *et al.* (2012) found lower clinical pregnancy (50.5% *vs*. 19.6%) and live birth rates (44.3% *vs*. 16.5%) with IVM, along with a significantly reduced risk of OHSS (8.2% *vs*. 0%).

**Table 1:** A comparison of IVM and IVF success rates.

Study	ART	Priming	Stage of embryo transferred	Clinical pregnancy (%)	Live birth (%)	OHSS(%)
Child (2002)	IVM	hCG	Days 2 or 3	26.2	15.9	0
	IVF	-	Days 2 or 3	38.3	26.2	11.2
Gremeau *et al*. (2012)	IVM	hCG	Days 2 or 3	19.6	16.5	0
	IVF	-	Days 2, 3 or 5	50.5	44.3	8.2
Das *et al*. (2014)	IVM	hCG	Days 2 or 3	32.4	23.5	0
	IVF	-	Days 2 or 3	45.8	40.7	8.3
Shavit *et al*. (2014)	IVM	Unstimulated or FSH + hCG	Days 2 or 3	25	13.3	0
	IVF	-	Days 2 or 3	40	28.8	0
Walls *et al*. (2015), fresh embryo transfer	IVM	FSH	Blastocyst (single)	29.7	18.8	0
	IVF	-	Blastocyst (single)	36.2	31	7.1
Walls *et al*. (2015), frozen embryo transfer	IVM	FSH	Blastocyst (single)	35.5	33.9	-
	IVF	-	Blastocyst (single)	36.8	29.9	-
Ho *et al*. (2019)	IVM	FSH + hCG	Days 2 or 3	48.5	36.5	0
	IVF	-	Days 2 or 3	56.6	40.8	3.5
Vuong *et al*. (2020), RCT	IVM	FSH + LH	Day 3	50.5	35.2	0
	IVF	-	Day 3	56.4	43.2	0.7
Zheng *et al*. (2022), RCT	IVM	Unstimulated	Blastocyst (single)	41.1	28	0
	IVF	-	Blastocyst (single)	50.7	37.5	6.3

Both of these initial studies utilized fresh transfers of multiple IVM cleavage-stage embryos (averaging 3.2 and 1.9 embryos, respectively) to compensate for their reduced developmental competence. This approach led to multiple pregnancy rates as high as 40% (Child, 2002; Gremeau *et al.*, 2012). These findings were further supported by additional retrospective studies involving cleavage-stage embryo transfers, which reported multiple pregnancy rates exceeding 30% (Das *et al.*, 2014; Ho *et al.*, 2019). However, these rates were comparable to those in the IVF control groups, suggesting that the high incidence of multiple pregnancies was more related to the transfer strategy employed rather than being specific to the IVM technology itself.

The shift in reproductive medicine toward transferring fewer embryos with higher developmental potential also extended to IVM. Walls *et al.* (2015) were the first to compare IVM and IVF outcomes after the transfer of a single blastocyst. In their study, which involved priming IVM cycles with FSH, they reported similar blastocyst formation rates (45% *vs*. 46%), usable blastocyst rates (38% *vs*. 40%), and a comparable proportion of cycles with complete failure of blastocyst development (16% *vs*. 14%) between IVM and IVF. However, IVM cycles yielded fewer total blastocysts (3.0 *vs*. 4.6), and the fresh transfer of a single blastocyst led to significantly lower live birth rate in IVM (18.8% *vs*. 31%).

Interestingly, a subset of IVM cycles involved freezing all blastocysts due to inadequate endometrial conditions for fresh transfer. When these frozen embryos were later transferred, similar live birth rates were observed between IVM and IVF (29.9% *vs*. 33.9%) (Walls *et al.*, 2015), suggesting that poor endometrial conditions may contribute, at least in part, to the lower success rates observed with IVM. In a subsequent study, Zheng *et al.* (2022) conducted the first RCT comparing IVM and IVF after a freeze-all strategy and single blastocyst transfer, this time without any priming in IVM cycles. Their design yielded markedly different outcomes between IVM and IVF, with IVM showing a lower rate of vitrified blastocysts (27.2% *vs*. 46.5%), a higher proportion of cycles with no available blastocysts (36.7% *vs*. 3.8%) and reduced live birth rates (28% *vs*. 37.5%). This highlights the potential role of FSH priming in improving the developmental competence of IVM embryos.

With the advent of embryo freezing, each oocyte retrieval cycle has the potential to result in multiple embryo transfers, making the cumulative live birth rate arguably the most clinically meaningful measure of success. This is particularly relevant for IVM, which tends to result in a lower number of good-quality embryos compared to IVF (Das *et al.*, 2014; Ho *et al.*, 2019). In light of this, several studies have consistently reported inferior cumulative live birth rates for IVM compared to IVF, with differences ranging from 10.5% to 18.6%, even when employing newer protocols like CAPA-IVM (Walls *et al.*, 2015; Ho *et al.*, 2019; Vuong *et al.*, 2020; Zheng *et al.*, 2022).

Overall, few studies have examined the differential success rates between IVM and IVF over the past 20 years, and those that do exist are quite heterogeneous regarding hormonal priming, laboratory protocols, and the stage of embryos transferred. Even fewer studies are RCTs. The available evidence suggests that IVM continues to result in lower-quality embryos and reduced live birth rates compared to IVF, although the cumulative difference may be as small as 10.5%. Whether a reduction in success rates of approximately 10% is an acceptable trade-off for the benefits of IVM, such as reduced costs, shorter treatment times, and avoidance of the unpleasant side effects associated with gonadotropins, remains an important topic for further discussion.

## IVM SAFETY

The consistently lower embryo quality observed in IVM combined with the additional culture steps required in the laboratory raises concerns about whether genetic and epigenetic factors in IVM embryos, while still compatible with live birth, could potentially impact the long-term health of children born through this technique.

Human oocytes undergo significant remodeling of their methylation and genomic imprinting patterns during oocyte growth (Yan *et al.*, 2021), which may be hypothetically compromised by disruption of this process *in vitro*, affecting the epigenetic safety of the offspring. However, studies have shown that IVM does not significantly alter methylation patterns in imprinted genes when compared to oocytes matured *in vivo* (Kuhtz *et al.*, 2014). Furthermore, blastocysts derived from IVM treatment in PCOS patients did not show any significant differences in methylation patterns or the expression of key epigenetic regulators compared to blastocysts from IVF-treated PCOS patients (Saenz-de-Juano *et al.*, 2019). Finally, bisulfite sequencing analysis of chorionic villus and cord-blood samples from children conceived through IVM revealed no significant differences in the methylation of key developmentally relevant genomic loci (Pliushch *et al.*, 2015). Overall, the available data suggests that the epigenetic stability of the offspring is not adversely affected by IVM.

Another primary concern relates to the chromosomal integrity of IVM embryos. Although most aneuploidies do not result in live births, certain chromosomal abnormalities can still manifest in viable offspring (Capalbo *et al.*, 2022). To investigate this issue, Zhang *et al.* (2010b) analyzed chromosomal abnormality rates in cleavage-stage embryos derived from both IVM and IVF using FISH on chromosomes 13, 15, 16, 18, 21, 22, X, and Y. The results revealed no significant difference in chromosomal abnormality rates between IVM and IVF embryos (58.7% *vs*. 57.4%, respectively). However, the study also found that the incidence of chromosomal abnormalities increased with the time required for oocyte maturation. Specifically, IVM embryos derived from oocytes that took 48 hours to reach the MII stage exhibited a higher frequency of abnormalities compared to those that matured within 24 hours. This suggests that the timing of oocyte maturation is a critical factor to consider when selecting IVM embryos for transfer. Similarly, Requena *et al.* (2009) reported aneuploidy rates of 60% in cleavage-stage embryos from IVM cycles compared to 33% in IVF cycles, although this difference was not statistically significant.

Later studies have employed advanced genetic methodologies, such as comparative genomic hybridization (aCGH) and next-generation sequencing (NGS), to analyze aneuploidies across all chromosomes in IVM-derived cleavage-stage embryos, rather than focusing on a specific set of chromosomes. These technologies enable the detection of a broader range of aneuploidies, previously undetectable with FISH analysis (ESHRE PGT-SR/PGT-A Working Group *et al.*, 2020). Notably, these studies reported comparable incidence of aneuploidies between IVM and IFV embryos, with rates ranging from 28% to 55.6% (Spits *et al.*, 2015; Li *et al.*, 2021).

When assessing the health of IVM offspring, several studies have consistently shown no significant differences in perinatal and obstetric outcomes compared to IVF (Cha *et al.*, 2005; Mikkelsen, 2005; Shu-Chi *et al.*, 2006; Söderström-Anttila *et al.*, 2006; Buckett *et al.*, 2007; Fadini *et al.*, 2012; Roesner *et al.*, 2017; Mostinckx *et al.*, 2019; Yu *et al.*, 2019; Belva *et al.*, 2020). These studies, with follow-up periods extending up to 2 and 7.5 years, evaluated key parameters such as birth weight, gestational age at delivery, sex ratio, developmental milestones (assessed via Bayley Scales), Apgar scores, pregnancy complications, congenital abnormalities, psychomotor and neuropsychological development, and karyotype. Notably, Foix-L’Hélias *et al.* (2014) reported an increase in average birth weight, length, and head circumference among female infants conceived via IVM compared to those conceived via IVF. It is noteworthy that some studies properly matched IVM patients with PCOS to IVF patients with PCOS (Cha *et al.*, 2005; Mostinckx *et al.*, 2019; Yu *et al.*, 2019; Belva *et al.*, 2020), while others did not account for the specific PCOS background (Shu-Chi *et al.*, 2006; Söderström-Anttila *et al.*, 2006; Buckett *et al.*, 2007; Foix-L’Hélias *et al.*, 2014; Roesner *et al.*, 2017), which could be an influencing factor. However, a recent meta-analysis, as well as findings from the first RCT comparing IVM and IVF, confirmed no significant differences in perinatal outcomes between the two techniques, reaffirming the safety of IVM (Vuong *et al.*, 2020; Strowitzki *et al.*, 2021).

Overall, the current evidence supports the notion that IVM is a safe treatment, showing similar rates of methylation at epigenetically relevant loci, chromosomal abnormalities in embryos, and obstetric/perinatal outcomes when compared to IVF. However, it is important to recognize that the available research is still limited and highly heterogeneous, particularly concerning the IVM protocols used and the study populations involved. To enhance the understanding of IVM safety, future research should: i) go beyond analyzing DNA methylation and consider other epigenetic factors such as histone modifications and chromatin remodeling; ii) assess chromosomal integrity using state-of-the-art procedures, as trophectoderm biopsy from blastocysts; and iii) focus specifically on PCOS patients, allowing for a more accurate comparison of ART procedures without the confounding effects of underlying infertility conditions.

## CONCLUSIONS AND PERSPECTIVES

Despite being one of the earliest ART explored, IVM has historically remained on the sidelines of mainstream IVF, with few significant innovations over the last five decades. However, recent scientific advances in the understanding of oocyte physiology have revitalized efforts to optimize IVM protocols. Innovations such as biphasic CAPA-IVM systems and the use of OSCs derived from iPSCs demonstrate that mimicking the native follicular microenvironment can significantly enhance oocyte competence and clinical outcomes.

Nevertheless, IVM still faces notable challenges. Despite improvements, embryo developmental potential and cumulative live birth rates remain slightly lower compared to standard IVF. Additionally, the technical demands of oocyte retrieval and laboratory culture require specialized expertise and resources, limiting its widespread adoption. While current data support the genetic and epigenetic safety of IVM offspring, longer-term and larger-cohort studies are needed to fully validate these findings.

Future innovative strategies may involve the integration of biphasic maturation systems, OSCs, microfluidic platforms, and dynamic three-dimensional (3D) culture models to simulate the dynamic follicular environment more accurately. Additionally, training IVF professionals and educating both staff and patients on the unique benefits of IVM could be key steps toward broader acceptance and implementation of this technique.

Ultimately, IVM holds the potential to expand access to ART by offering a safer, more affordable, and less invasive treatment option, particularly for patients with high ovarian reserve, PCOS, or those requiring fertility preservation. As research continues to bridge the gap between laboratory culture and physiological conditions, IVM may yet fulfill its early promise as a mainstream alternative in reproductive medicine.
